# Synthesis of a new biological response modifier thyrnosin β_4_ analogue and its restorative effect on depressed

**DOI:** 10.1155/S096293519200019X

**Published:** 1992

**Authors:** Takashi Abiko, Hiroshi Sekino

**Affiliations:** Kidney Research Laboratory Kojinkai 1-6 Tsutsujigaoka 2-chome Miyagino-ku Sendai 980 Japan

## Abstract

Thymosin β_4_ is a polypeptide isolated from thymosin fraction 5. This peptide exhibits important activities in the regulation and differentiation of thymus-dependent lymphocytes. An analogue of thymosin β_4_, [Phe(4F)^12^] deacetyl- thymosin β_4_, was synthesized by a solution method, followed by deprotection with 1 M trifluoromethanesulphonic acid (TFMSA)-thioanisole (molar ratio, 1:1) in trifluoroacetic acid (TFA) in the presence of dimethlselenium. Finally, the deprotected peptide was incubated with dithiothreitol to reduce sulphoxide on the methionine side chain. The synthetic [Phe(4F)^12^]deacetyl-thymosin β_4_ was found to have a restoring effect on the impaired blastogenic response of T-lymphocytes isolated from uraemic patients with recurrent infectious diseases. This analogue exhibited stronger restorative activity than that of our synthetic deacetyl-thymosin β_4_.


Mediators of Inflammation, 1, 113–119 (1992)

### Synthesis of a new biological response modifier thymosin β4 analogue and its restorative effect on depressed

**Takashi Abiko[CA] and Hiroshi Sekino**

Kidney Research Laboratory, Kojinkai, 1-6 Tsutsujigaoka 2-chome, Miyagino-ku, Sendai 980, Japan

[CA] Corresponding Author

**THYMOSIN $\beta_4$ is a polypeptide isolated from thymosin fraction 5. This peptide exhibits important activities in the regulation and differentiation of thymus-dependent lymphocytes. An analogue of thymosin $\beta_4$, [Phe(4F)$^{12}$]deacetyl-thymosin $\beta_4$, was synthesized by a solution method, followed by deprotection with 1 M trifluoromethanesulphonic acid (TFMSA)-thioanisole (molar ratio, 1:1) in trifluoroacetic acid (TFA) in the presence of dimethylselenium. Finally, the deprotected peptide was incubated with dithiothreitol to reduce sulphoxide on the methionine side chain. The synthetic [Phe(4F)$^{12}$]deacetyl-thymosin $\beta_4$ was found to have a restoring effect on the impaired blastogenic response of T-lymphocytes isolated from uraemic patients with recurrent infectious diseases. This analogue exhibited stronger restorative activity than that of our synthetic deacetyl-thymosin $\beta_4$.**

**Key words:** Dithiothreitol reduction, Impaired blastogenic response, [Phe(4F)$^{12}$]deacetyl-thymosin $\beta_4$ synthesis, Restoring effect, Trifluoromethanesulphonic acid deprotection, Uraemic patient

### Introduction

The impairment of immunological responsiveness in uraemic patients is well documented.[1,2] All aspects of the immune response appear to be affected by the uraemic state. The numbers, subpopulations and reactivities of circulating lymphocytes may be altered by uraemia.[2,3] This impairment has been implicated in easy susceptibility to infections and increased incidence of malignancy. In addition, various investigators have reported severe structural changes in the lymph nodes[4] and thymus glands of uraemic patients and animals.[5]

The thymus plays an essential role in the development and maintenance of cellular immune competence.[6] Recent evidence suggests that the thymus produces biologically active peptides which are responsible for the differentiation and functional maturation of precursor T-lymphocytes.[7]

Thymosin $\beta_4$, an N-terminal acetylated peptide containing 43 amino acid residues, was first isolated from calf thymus by Low *et al.*[8] who determined its amino acid sequence. This peptide exhibits important activities in the regulation and differentiation of thymus-dependent lymphocytes.[8] It also inhibits the migration of macrophages, and exerts biological effects on the hypothalamus and pituitary.

In our previous papers,[9–11] we reported syntheses of deacetyl-thymosin $\beta_4$ and its fragments, and showed that the synthetic deacetyl-thymosin $\beta_4$ and some of the fragments could have restorative effects on the impaired cell-mediated immunological functions.

We also noticed that the acetyl group at the N-terminal serine residue of thymosin $\beta_4$, is not required for the restorative effect on the impaired cell-mediated immunological functions.[9]

In our preceding paper,[10] we also concluded that the two portions of the amino acid sequence of thymosin $\beta_4$, -Lys-Leu-Lys-Lys-Thr-Glu-Thr-Gln-Glu-Lys-Asn- (positions 16–26) and -Lys-Glu-Thr-Ile-Glu-Gln-Lys-Gln- (positions 31–39), are important structures in thymosin $\beta_4$ for the restoration activity on impaired cell-mediated immunological functions and thymosin $\beta_4$ was effective at lower concentration than those two synthetic fragments. So other portions of thymosin $\beta_4$ may also be necessary for the full activity.

In 1989, Maeda *et al.*[12] reported that one of the Leu-enkephalin analogues, [Phe(4F)$^4$]Leu-enkephalin, showed stronger activity in the guinea-pig ileum and mouse vas deferens assays as compared with Leu-enkephalin.

**Abbreviations:** Z, benzyloxycarbonyl; OBzl, benzyl ester; Bzl, benzyl; Boc, *tert*-butoxycarbonyl; Troc, $\beta,\beta,\beta$-trichloroethoxycarbonyl; Su, N-hydroxysuccinimide; NMM, N-methylmorpholine; OSu, N-hydroxy-succinimide ester; EDTA, ethylenediaminetetraacetic acid; DMF, dimethyl-formamide; DMSO, dimethylsulfoxide; AcOH, acetic acid; MeOH, methanol; EtOAc, ethyl acetate; RPMI, Rosewell Park Memorial Institute; Phe(4F), *p*-fluorophenylalanine; HPLC, high-performance liquid chromatography; DEAE, diethylaminoethyl. PHA, phytohaemagglutinin.

© 1992 Rapid Communications of Oxford Ltd

This result prompted us to synthesize a thymosin $\beta_4$ analogue containing a fluorinated aromatic ring. This paper deals with the synthesis of [Phe(4F)[12]]-deacetyl-thymosin $\beta_4$, and an examination of the immunological effect of this analogue and our synthetic deacetyl-thymosin $\beta_4$[9] on the impaired blastogenic response of T-lymphocytes of uraemic patients.

### Materials and Methods

General experimental procedures used were essentially the same as described in the previous papers.[13,14] Preparations of protected intermediates were repeated several times in order to obtain sufficient quantities for the next step. Melting points are uncorrected. Optical rotations were measured with an Atago Polax machine (cell length: 10 cm). The amino acid compositions of the acid and enzymatic hydrolysates were determined with an Hitachi type 835-50 amino acid analyser. Solutions were concentrated in a rotary evaporator under reduced pressure at a temperature of 30–45°C.

Boc groups of the protected peptides were removed by TFA-anisole treatment. The resulting amino components were chromatographed on silica gel plates (Kieselgel G, Merck) and $R_F^1$ values refer to the following solvent system: $CHCl_3$-MeOH-$H_2O$ (8:3:1). The final product corresponding to the entire amino acid sequence of [Phe(4F)[12]]deacetyl-thymosin $\beta_4$ was chromatographed on cellulose plates (Merck). $R_F^2$ value refers to BuOH-AcOH-$H_2O$ (5:1:1) and $R_F^3$ value refers to BuOH-pyridine-AcOH-$H_2O$ (30:20:6:24). Troc-$NHNH_2$ was purchased from Kokusan Chemical Works Ltd., Japan. Papain (No. P-3125) and leucine aminopeptidase (No. L-9876) were purchased from Sigma Chemical Co.

*Preparation of peptides:*

Boc-Lys(Z)-Phe(4F)-NHNH-Troc **[I]**. Boc-Phe(4F)-NHNH-Troc (1.9 g) was treated with TFA-anisole (19 ml–3.8 ml) in an ice bath for 40 min, and TFA was then removed by evaporation. The residue was washed with *n*-hexane, dried over KOH pellets *in vacuo* for 2 h, and then dissolved in DMF (10 ml) containing NMM (0.6 ml). To this solution, Boc-Lys(Z)-OSu (2.1 g) was added, and the mixture was stirred at room temperature for 7 h. The mixture was extracted with EtOAc and then washed successively with 5% $NaHCO_3$, $H_2O$, 5% citric acid and $H_2O$, dried over $MgSO_4$, and concentrated *in vacuo*. The residue was reprecipitated from EtOAc with *n*-hexane. Yield 2.2 g (79%), m.p. 81–87°C, $[\alpha]_D^{21}$ −15.3° (c = 1.0, DMF), $R_F^1$ 0.78, single ninhydrin-positive spot. Anal. Calcd. for $C_{31}H_{39}Cl_3FN_5O_8H_2O$: C, 54.04; H, 6.00; N, 10.16. Found; C, 53.81; H, 6.29; N, 10.42.

Boc-Glu(OBzl)-Lys(Z)-Phe(4F)-NHNH-Troc **[II]**. This compound was prepared essentially in the same manner as described for the preparation of **I** using **I** (2.5 g) and Boc-Glu(OBzl)-OSu (1.7 g). The product was reprecipitated from EtOAc with ether. Yield 2.7 g (75%), m.p. 103–109°C, $[\alpha]_D^{21}$ −14.8° (c = 1.0, DMF), $R_F^1$ 0.82, single ninhydrin-positive spot. Anal. Calcd. for $C_{43}H_{52}Cl_3FN_6O_{11}$ $2H_2O$: C, 52.15; H, 5.70; N, 8.49. Found: C, 52.03; H, 6.08; N, 8.71.

Boc-Ile-Glu(OBzl)-Lys(Z)-Phe(4F)-NHNH-Troc **[III]**. This compound was prepared from **II** (2.5 g) and Boc-Ile-OSu (903 mg) essentially as described for the preparation of **I**. The product was reprecipitated from EtOAc with ether. Yield 2 g (71%), m.p. 130–136°C, $[\alpha]_D^{21}$ −8.7° (c = 1.0, DMF), $R_F^1$ 0.84, single ninhydrin-positive spot. Anal. Calcd. for $C_{49}H_{63}Cl_3FN_7O_{12}$ $2H_2O$: C, 53.34; H, 6.12; N, 13.73. Found: C, 53.11; H, 6.49; N, 13.80.

Boc-Glu(OBzl)-Ile-Glu(OBzl)-Lys(Z)-Phe(4F)-NHNH-Troc **[IV]**. This compound was prepared from **III** (1.6 g) and Boc-Glu(OBzl)-OSu (683 mg) essentially as described for the preparation of **I**. The product was reprecipitated from acetone with ether. Yield 1.4 g (74%), m.p. 103–110°C, $[\alpha]_D^{21}$ −12.6° (c = 1.0, DMF), $R_F^1$ 0.84, single ninhydrin-positive spot. Anal. Calcd. for $C_{61}H_{76}Cl_3FN_8O_{15}$ $3H_2O$: C, 54.65; H, 6.17; N, 8.36. Found: C, 54.42; H, 6.41; N, 8.59.

Boc-Ala-Glu(OBzl)-Ile-Glu(OBzl)-Lys(Z)-Phe(4F)-NHNH-Troc **[V]**. This compound was prepared from **IV** (1.3 g) and Boc-Ala-OSu (3.1 g) essentially as described for the preparation of **I**. The product was reprecipitated from EtOAc with ether. Yield 1.1 g (79%), m.p. 132–139°C, $[\alpha]_D^{21}$ −11.4° (c = 1.0, DMF), $R_F^1$ 0.85, single ninhydrin-positive spot. Anal. Calcd. for $C_{64}H_{81}Cl_3FN_9O_{16}$ $4H_2O$: C, 53.80; H, 6.27; N, 8.82. Found: C, 53.60; H, 6.37; N, 8.45.

Boc-Met(O)-Ala-Glu(OBzl)-Ile-Glu(OBzl)-Lys(Z)-Phe(4F)-NHNH-Troc **[VI]**. Compound **V** (1 g) was treated with TFA-anisole (10 ml–2 ml) as described above. Boc-Met(O)-Su (272 mg) was added to a solution of this product in DMF (10 ml), followed by NMM to keep the solution slightly alkaline. After 8 h at room temperature, the reaction mixture was poured into 5% $NaHCO_3$ with stirring. The precipitate thereby formed was washed successively with 5% $NaHCO_3$, $H_2O$, citric acid and $H_2O$. The dried product was reprecipitated from MeOH with ether. Yield 986 mg (88%), m.p. 130–137°C, $[\alpha]_D^{21}$ −7.3° (c = 1.0, DMF), $R_F^1$ 0.80, single ninhydrin-positive spot. Anal. Calcd. for $C_{69}H_{90}Cl_3FN_{10}O_{18}S$ $5H_2O$: C, 51.96; H, 6.32; N, 8.78. Found: C, 51.71; H, 6.54; N, 8.97.

Boc - Asp(OBzl) - Met(O) - Ala - Glu(OBzl) - Ile - Glu(OBzl)-Lys(Z)-Phe(4F)-NHNH-Troc **[VII]**. This compound was prepared from **VI** (798 mg) and Boc-Asp(OBzl)-OSu (232 mg) essentially as described for the preparation of **VI**. The dried product was reprecipitated from hot EtOAc. Yield 765 mg (86%), m.p. 150–158°C, $[\alpha]_D^{21}$ $-11.4°$ (c = 1.0, DMF), $R_F^1$ 0.86, single ninhydrin-positive spot. Anal. Calcd. for $C_{80}H_{101}Cl_3FN_{11}O_{21}S$ $4H_2O$: C, 53.92; H, 6.17; N, 8.65. Found: C, 53.75; H, 6.44; N, 8.59.

Boc - Asp(OBzl) - Met(O) - Ala - Glu(OBzl) - Ile - Glu(OBzl)-Lys(Z)-Phe(4F)-NHNH$_2$ **[VIII]**. Compound **VII** (594 mg) in a mixture of AcOH (6 ml) and DMF (6 ml) was treated with zinc dust (220 mg) at 4°C for 12 h. The solution was filtered, the filtrate was concentrated *in vacuo*, and the residue was treated with 3% EDTA and then with $NaHCO_3$ to adjust the pH to neutral. The resulting powder was washed with $H_2O$ and reprecipitated from DMF with $H_2O$. Yield 487 mg (91%), m.p. 178–186°C, $[\alpha]_D^{21}$ $-19.4°$ (c = 1.0, DMF), $R_F^1$ 0.76, single hydrazine-test-positive spot. Anal. Calcd. for $C_{77}H_{100}FN_{11}O_{19}S$ $4H_2O$: C, 57.56; H, 6.78; N, 9.59. Found: C, 57.46; H, 6.91; N, 9.41.

Boc - Asp(OBzl) - Met(O) - Ala - Glu(OBzl) - Ile - Glu(OBzl)-Lys(Z)-Phe(4F)-Asp(OBzl)-Lys(Z)-Ser(Bzl)-Lys(Z)-Leu - Lys(Z) - Lys(Z) - Thr(Bzl) - Glu(OBzl) - Thr(Bzl) - Gln - Glu(OBzl) - Lys(Z) - Asn - Pro - Leu - Pro - Ser(Bzl) - Lys(Z) - Glu(OBzl) - Thr(Bzl) - Ile - Glu(OBzl) - Gln - Glu(OBzl) - Lys(Z) - Gln - Ala - Gly - Gu(OBzl) - Ser(Bzl) - OBzl **[IX]**. This compound was prepared as follows: Boc - Asp(OBzl) - Lys(Z) - Ser(Bzl) - Lys(Z) - Leu - Lys(Z) - Lys(Z) - Thr(Bzl) - Glu(OBzl) - Thr(Bzl) - Gln - Glu(OBzl) - Lys(Z) - Asn - Pro - Leu - Pro - Ser(Bzl) - Lys(Z) - Glu(OBzl) - Thr(Bzl) - Ile - Glu(OBzl) - Gln - Glu(OBzl) - Lys(Z) - Gln - Ala - Gly - Glu(OBzl) - Ser(Bzl)-OBzl (151 mg) was treated with TFA-anisole (2 ml–0.4 ml) as usual and dissolved in DMF-DMSO (1:1, 3 ml) containing NMM (0.03 ml). The azide [prepared from 121 mg of **VIII** (3 eq)] in DMF-DMSO (1:1, 2 ml) and NMM (0.042 ml) were added to the above ice-chilled solution and the mixture was stirred at −10°C for 36 h until the solution became ninhydrin-negative. After addition of a few drops of AcOH, the mixture was poured into 5% citric acid with stirring. The resulting powder was washed with 5% citric acid, $H_2O$ and MeOH and purified by gel filtration on Sephadex LH-60 using DMSO containing 3% $H_2O$ as an eluent. The desired fractions (each 5 ml, tube nos 45–56) were combined, the solvent was evaporated off, and the residue was treated with MeOH to afford a powder. Yield 139 mg (75%), m.p. 184–195°C, $[\alpha]_D^{21}$ $-18.3°$ (c = 1.0, DMSO), $R_F^1$ 0.82, single ninhydrin-positive spot. Anal. Calcd. for $C_{380}H_{476}FN_{51}O_{89}S$ $13H_2O$: C, 61.12; H, 6.78; N, 9.57. Found: C, 60.85; H, 6.96; N, 9.58. Amino acid ratios in a 6 N HCl hydrolysate: Gly 1.00, Leu 2.09, Ile 0.95, Met(O) 0.94, Ala 2.04, Pro 1.91, Phe(4F) 0.93, Ser 2.89, Thr 2.91, Glu 10.86, Asp. 2.94, Lys 7.95 (recovery of Gly 86%).

Boc - Ser(Bzl) - Asp(OBzl) - Lys(Z) - Pro - Asp(OBzl)-Met(O)-Ala-Glu(OBzl)-Ile-Glu(OBzl)-Lys(Z)-Phe(4F)-Asp(OBzl)-Lys(Z)-Ser(Bzl)-Lys(Z)-Leu-Lys(Z)-Lys(Z)-Thr(Bzl) - Glu(OBzl) - Thr(Bzl) - Gln - Glu(OBzl) - Lys(Z)-Asn - Pro - Leu - Pro - Ser(Bzl) - Lys(Z) - Glu(OBzl)-Thr(Bzl) - Ile - Glu(OBzl) - Gln - Glu(OBzl) - Lys(Z) - Gln-Ala-Gly-Glu(OBzl)-Ser(Bzl)-OBzl **[X]**. Compound **IX** (93 mg) was treated with TFA-anisole (2 ml–0.4 ml) as usual and dissolved in DMF-DMSO (1:1, 3 ml) containing NMM (0.015 ml). The azide [prepared from 33 mg of Boc-Ser(Bzl)-Asp(OBzl)-Lys(Z)-Pro-NHNH$_2$[9] (3 eq)] in DMF-DMSO (1:1, 2 ml) and NMM (0.05 ml) were added to the above ice-chilled solution and the mixture was stirred at −10°C for 36 h. Additional azide (1 eq) in DMF-DMSO (1:1, 2 ml) and NMM (0.016 ml) was then added and stirring was continued for an additional 24 h until the solution became ninhydrin-negative. After addition of a few drops of AcOH, the mixture was poured into 5% citric acid with stirring. The resulting powder was washed with 5% citric acid, $H_2O$ and MeOH. The product was reprecipitated from DMSO with MeOH. Yield 65 mg (63%), m.p. 181–192°C, $[\alpha]_D^{21}$ $-23.8°$ (c = 1.0, DMSO), $R_F^1$ 0.86, single ninhydrin-positive spot. Anal. Calcd. for $C_{420}H_{523}FN_{56}O_{98}S$ $17H_2O$: C, 60.92; H, 6.78; N, 9.47. Found: C, 60.57; H, 6.83; N, 9.43. Amino acid ratios in a 6N HCl hydrolysate: Gly 1.00, Leu 1.97, Ile 1.94, Met(O) 0.92, Ala 2.05, Pro 2.88, Phe(4F) 0.90, Ser 3.89, Thr 2.91, Glu 11.03, Asp 3.94, Lys 8.98 (recovery of Gly 84%).

H-Ser-Asp-Lys-Pro-Asp-Met-Ala-Glu-Ile-Glu-Lys-Phe(4F)-Asp-Lys-Ser-Lys-Leu-Lys-Lys-Thr-Glu-Thr-Gln-Glu-Lys-Asn-Pro-Leu-Pro-Ser-Lys-Glu-Thr-Ile-Glu-Gln-Glu-Lys-Gln-Ala-Gly-Glu-Ser-OH (corresponding to [Phe(4F)[12]]deacetyl-thymosin $\beta_4$ **[XI]**. The protected tritetracontapeptide ester **X** (50 mg) was treated with 1 M TFMSA-thioanisole in TFA (2 ml) in the presence of $Me_2Se$ (50 $\mu$l) in an ice bath for 120 min, then dry ether was added. The resulting powder was collected by centrifugation, dried over KOH pellets for 2 h and dissolved in 1 N AcOH (5 ml). After being stirred with Amberlite IRA-400 (acetate form, approximately 1 g) for 30 min, the solution was adjusted to pH 6.0 with 1 N AcOH and the solution was lyophilized. The residue was dissolved in $H_2O$ (5 ml). The solution, after addition of dithiothreitol (10 mg), was incubated at 60°C for 36 h. The solvent was evaporated *in vacuo* and the residue was dissolved in a small amount of

1% AcOH and then applied to a column of Sephadex G-25 (2.4 × 93 cm), which was eluted with 1% AcOH. Individual fractions (4 ml each) were collected and the absorbance at 260 nm was determined for each fraction. The fractions corresponding to the first main peak (tube nos 52–61) were combined and the solvent was removed by lyophilization. The residue was dissolved in $H_2O$ (2 ml) and the solution was applied to a column of DEAE-cellulose (Brown, 2.4 × 50 cm), eluted with a linear gradient of 400 ml each of $H_2O$ and 0.08 M $NH_4HCO_3$ buffer at pH 7.8. Individual fractions (4 ml each) were collected and the absorbance at 260 nm was determined. The main peak present in the gradient eluates (tube nos 69–77) were combined and the solvent was condensed to approximately 2 ml. This solution was then applied to a column of Sephadex G-25 (2.4 × 93 cm), which was eluted with 1% AcOH. The desired fractions (4 ml each, tube nos 50–59) were collected and the solvent was removed by lyophilization to give a fluffy powder. Yield 7.1 mg (24%) $[\alpha]_D^{21}$ −79.3° (c = 0.3, 1 N AcOH), $R_F^2$ 0.01, $R_F^3$ 0.08, single ninhydrin- and chlorine-tolidine-positive spot. The synthetic peptide exhibited a single spot on a paper electrophoresis: Toyo Roshi No. 51 (2 × 40 cm) at pH 7.1 pyridinium-acetate buffer; mobility, 1.5 cm from the origin toward the anode, after running at 2 mA, 600 V for 80 min. The synthetic peptide exhibited a single peak on HPLC using an analytical Nucleosil 5C18 column (4 × 150 mm) at a retention time of 15.48 min, when eluted with a gradient of acetonitrile (20–45%) in 0.1% TFA at a flow rate of 1 ml/min (Fig. 3). Amino acid ratios in a 6 N HCl hydrolysate: Gly 1.00, Leu 2.04, Ile 1.97, Met 0.89, Ala 2.05, Pro 2.84, Phe(4F) 0.92, Ser 3.87, Thr 2.89, Glu 10.96, Asp 3.88, Lys 9.02 (recovery of Gly 86%). Amino acid ratios in papain plus leucine aminopeptidase digest: Gly 1.00, Leu 2.06, Ile 2.03, Met 0.87, Ala 2.01, Pro 2.85, Phe(4F) 0.94, Ser 3.90, Thr 2.91, Glu 7.89, Asp 2.95, Lys 3.92; Gln and Asn were not determined (recovery of Gly 84%).

*Patient selection:* Two uraemic patients who were suffering from recurrent infectious diseases were selected. Examination of the cellular immunocompetence of these patients revealed a significant decrease in blast formation by PHA. [$^3$H]-Thymidine incorporation values of these patients were 9081 and 10 748 cpm, respectively (normal values: 38 692–39 463 cpm). Venous blood was obtained from these uraemic patients for the blast formation test. Venous blood samples from three healthy donors were used as a control. HPLC was conducted with a Shimadzu LC-3A apparatus equipped with a Nucleosil 5C18 column.

*Blast formation test:* PHA-P (Difco) was added at optimal concentration of 1 $\mu$g/ml. T-lymphocytes were cultured in 0.2 ml of RPMI 1640 plus 10% foetal calf serum in microtitre plates (Falcon No. 3040). Then 0.02 ml (final 1 $\mu$g/ml) of PHA was added, with either the synthetic [Phe(4F)$^{12}$]deacetyl-thymosin $\beta_4$ or control peptide (deacetyl-thymosin $\beta_4$). Triplicate cultures of each combination of 5 × $10^5$ cells per well were incubated at 37°C in a humidified atmosphere at 5% $CO_2$ in air for 3 days. Before harvest (24 h), 1 $\mu$Ci of [$^3$H]-thymidine was added per culture. The amount of thymidine incorporation into DNA was measured in a scintillation counter (Table 1).

### Results

From a synthetic viewpoint, compared with our previous syntheses of deacetyl-thymosin $\beta_4$[9] and its fragments,[10,11] the thioanisole-mediated TFMSA deprotection procedure[13] was applied in the final step of the present synthesis instead of catalytic hydrogenation or hydrogen fluoride.

Our synthetic route to [Phe(4F)$^{12}$]deacetyl-thymosin $\beta_4$ is illustrated in Fig. 1, which shows three fragments selected as building blocks to

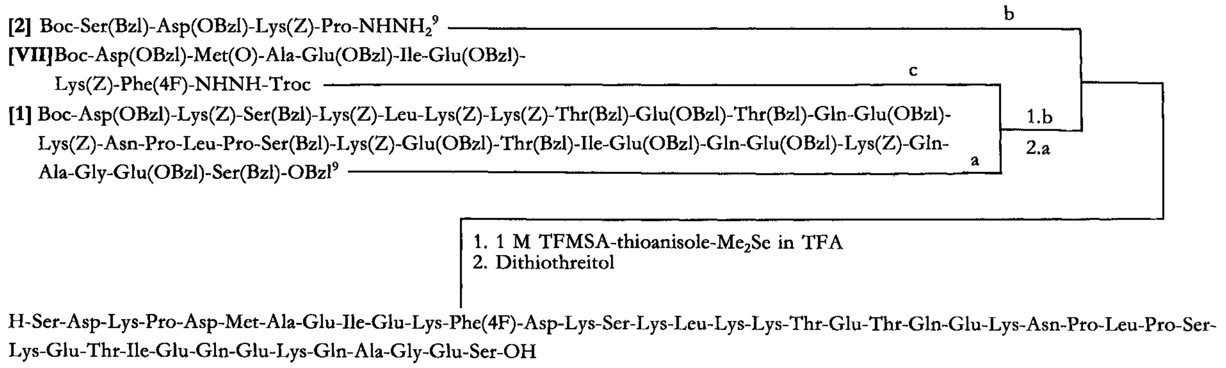

FIG. 1. Synthetic route to [Phe(4F)$^{12}$]deacetyl-thymosin $\beta_4$. a: TFA-anisole; b, azide; c, Zn-AcOH.

construct the entire amino acid sequence of [Phe(4F)[12]]deacetyl-thymosin $\beta_4$. Of these, two fragments, **[1]** and **[2]**, are those employed for our previous synthesis of deacetyl-thymosin $\beta_4$.[9] Thus, one fragment **[VII]**, which covers the area of sequence variation between deacetyl-thymosin $\beta_4$ and [Phe(4F)[12]]deacetyl-thymosin $\beta_4$, was newly synthesized.

Amino acid derivatives bearing protecting groups removable by 1 M TFMSA-thioanisole in TFA[13] were employed, i.e. Ser(Bzl), Thr(Bzl), Lys(Z), Glu(OBzl), Asp(OBzl) and Ser(Bzl)-OBzl. The Met residue was reversibly protected as its sulphoxide in order to prevent partial S-alkylation during the $N^{\alpha}$-TFA deprotection as well as partial air oxidation during the synthesis. The Boc group, removable by TFA, was adopted as a temporary $N^{\alpha}$-protecting group for every intermediate. The substituted hydrazine, Troc-$NHNH_2$,[15] was employed for the preparation of a fragment **[VII]**, Boc - Asp(OBzl) - Met(O) - Ala - Glu(OBzl) - Ile-Glu(OBzl)-Lys(Z)-Phe(4F)-NHNH-Troc, containing the Glu(OBzl) and Asp(OBzl) residues. This Troc group is known to be cleaved by zinc[15] in AcOH without affecting other functional groups.

Throughout the synthesis, the purity of every intermediate was checked by thin-layer chromatography, elemental analysis and amino acid analysis of acid hydrolysates. The analytical results were within $\pm 0.4\%$ of theoretical values in all cases.

The protected octapeptide fragment **[VII]**, Boc-(5-12)-NHNH-Troc, was prepared stepwise by the Su active ester procedure to yield Boc-Asp(OBzl)-Met(O)-Ala-Glu(OBzl)-Ile-Glu(OBzl)-Lys(Z)-Phe(4F)-NHNH-Troc **[VII]**, and the Boc groups of intermediates were removed by treatment with TFA-anisole prior to the next coupling reaction. The protected octapeptide fragment **[VII]** thus obtained was treated with zinc[15] in AcOH to remove the Troc group, and the zinc acetate was removed by treatment with EDTA to give the required hydrazide, Boc-(5-12)-$NHNH_2$ **[VIII]**, in an analytically pure form. The hydrazine test on the thin-layer chromatogram and elemental analysis data were consistent with homogeneity of the desired product. The three fragments were successively condensed by the azide procedure according to the route shown in Fig.1.

Every reaction was carried out in a mixture of DMF and DMSO and the amount of the acyl component was increased from three to four equivalents as the chain elongation progressed. Every product was purified either by precipitation from DMSO with MeOH or by gel filtration on Sephadex LH-60. Throughout the synthesis, Gly was used as a diagnostic amino acid in acid hydrolysis. By comparison of recovery of Gly with those newly incorporated amino acids, satisfactory incorporation of each fragment was ascertained.

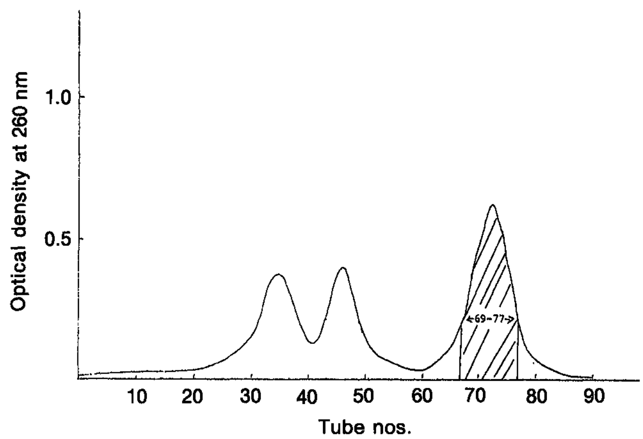

FIG. 2. Purification of synthetic [Phe(4F)[12]]deacetyl-thymosin $\beta_4$ by ion-exchange chromatography on a DEAE-cellulose column.

In the final step of the synthesis, the protected tritetracontapeptide ester was treated with 1 M TFMSA-thioanisole in TFA in the presence of $Me_2Se$, which was employed to facilitate acidic cleavage of protecting groups. The unprotected peptide was next precipitated with peroxide-free ether, converted to the corresponding acetate with Amberlite IRA-400 (acetate form) and then treated with 1 N $NH_4OH$ at pH 8.0 to reverse a possible N → O shift at the Ser and Thr residues. The Met (O) residue was reduced back to Met in two steps, firstly with thioanisole and $Me_2Se$ during the above acid treatment, and secondly with dithiothreitol during incubation of the unprotected peptide. The reduced product was purified by gel filtration on Sephadex G-25, followed by ion-exchange column

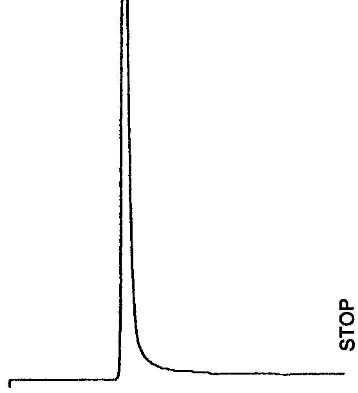

FIG. 3. HPLC of synthetic [Phe(4F)[12]]deacetyl-thymosin $\beta_4$.

**Table 1**. Effect of the synthetic deacetyl-thymosin $\beta_4$ and [Phe(4F)$^{12}$]deacetyl-thymosin $\beta_4$ on the impaired PHA stimulation of T-lymphocytes of uraemic patients

| Reaction mixture | No. of determ[illegible]tions | Dose of peptide ($\mu$g/ml) | [$^3$H]-thymidine incorporation (cpm) |
|---|---|---|---|
| A only | 3 | — | 406 ± 36 |
| B only | 3 | — | 39 077 ± 3 418 |
| C only | 3 | — | 9 915 ± 3 219 |
| Deacetyl-thymosin $\beta_4$ + C | 3 | 0.1 | 9 862 ± 3 197 |
| Deacetyl-thymosin $\beta_4$ + C | 3 | 1.0 | 19 628 ± 3 192 |
| [Phe(4F)$^{12}$]deacetyl-thymosin $\beta_4$ + C | 3 | 0.1 | 26 187 ± 3 248 |
| [Phe(4F)$^{12}$]deacetyl-thymosin $\beta_4$ + C | 3 | 1.0 | 30 119 ± 3 186 |
| H-Gly-Gly-Lys-OH + C | 3 | 1.0 | 9 628 ± 3 119 |

A: Normal lymphocytes, PHA(−); B: normal lymphocytes, PHA(+); C: uraemic patient's lymphocytes, PHA(+). All incubations were carried out at 37°C in a humidified atmosphere of 5% $CO_2$ in air for 12 h. The control, H-Gly-Gly-Lys-OH, was purchased from the Peptide Insitute Inc., Osaka.

chromatography on a DEAE-cellulose column with linear gradient elution using pH 7.8 $NH_4HCO_3$ buffer, followed by Sephadex G-25 column chromatography and the solvent was removed by lyophilization. The product exhibited a single spot (ninhydrin- and chlorine-tolidine-positive) on TLC in two different solvent systems and paper electrophoresis (pH 7.1 pyridinium acetate buffer). Its purity was further confirmed by amino acid analysis after acid hydrolysis and enzymatic digestion. The synthetic peptide exhibited a single peak on HPLC.

The immunological effect of the synthetic deacetyl-thymosin $\beta_4$[9] and [Phe(4F)$^{12}$]deacetyl-thymosin $\beta_4$ was examined by means of the lymphocyte stimulation test by PHA.[16] Responses of T-lymphocytes to mitogenic stimulation were lower in uraemic patients than those of normal persons. The *in vitro* effect of the two synthetic peptides on the impaired PHA response of T-lymphocytes from uraemic patients is shown in Table 1.

### Discussion

We have reported[9–11] evidence of impaired immune function in patients with chronic uraemia. This impairment is reflected in both *in vitro* and *in vivo* depressed cell-mediated immune function. Patients with chronic uraemia may have thymic atrophy. The thymus may show a marked reduction in lymphoid elements and extensive replacement infiltration with fat. Cystic degradation may be seen. These observations and findings suggested to us that the cell-mediated immune abnormalities seen in chronic uraemia might be attributable to thymic hormone deficiency. The test involves the *in vitro* incubation of patient lymphocytes with the synthetic peptides and the results of incubations with the [Phe(4F)$^{12}$]deacetyl-thymosin $\beta_4$ and deacetyl-thymosin $\beta_4$ are compared.

Comparison of the amount of [$^3$H]-thymidine incorporation into DNA shows that, in the case of the uraemic patients investigated, not only the synthetic deacetyl-thymosin $\beta_4$[9] but also the synthetic [Phe(4F)$^{12}$]deacetyl-thymosin $\beta_4$ exhibited restoring effect.

Interestingly, our synthetic [Phe(4F)$^{12}$]deacetyl-thymosin $\beta_4$ showed stronger restoring activity than that of our synthetic deacetyl-thymosin $\beta_4$. In this study, the strong electron withdrawing fluoride group on the *para* position of the aromatic ring results in an analogue that possesses stronger activity than that of the parent molecule.

Maeda *et al.* also reported[12] that [Phe(4F)$^4$] Leu-enkephalin showed stronger activity in the guinea-pig ileum and mouse vas deferens assays as compared with Leu-enkephalin. These results might suggest that modification of the Phe residue of thymosin $\beta_4$ could produce more potent analogues capable of a restorative effect on impaired blastogenic response of T-lymphocytes.

### References

1. Dobbelstein H. Immune System in Uremia. *Nephron* 1976; **17:** 409–414.
2. Traeger J, Trouraine JL, Navarro J, Freyria J, Contreas P. Immunodeficiency in Chronic Renal Insufficiency. *Proc Eur Dial Transplant Ass* 1980; **17:** 375–382.
3. Casciani CU, DeSimone C, Bonini S *et al.* Immunological Aspects of Chronic Uremia. *Kidney Int* 1978; **13 suppl:** 49–54.
4. Black MM, de Chabon A. Reactivity of Lymph Nodes in Azotemic Patients. *Am J Clin Pathol* 1964; **41:** 503–508.
5. Roskova J, Morrison AB. A Decrease in Cell-mediated Immunity in Uremia Associated with an Increase in Activity of Suppressor Cells. *Am J Pathol* 1976; **84:** 1–10.
6. Miller JFAP. Immunological Function of the Thymus. *Lancet* 1961; **ii:** 48–53.
7. Skotnicki AB. Thymic Hormones and Lymphokines. *Drugs of Today* 1989; **25:** 337–362.
8. Low TLK, Hu SK, Goldstein AL. Complete Amino Acid Sequence of Bovine Thymosin $\beta_4$: A thymic hormone that induces terminal deoxynucleotidyl transferase activity in thymocyte populations. *Proc Natl Acad Sci USA* 1981; **78:** 1162–1166.
9. Abiko T, Sekino H. Deacetyl-thymosin $\beta_4$: Synthesis and effect on the impaired peripheral T-cell subsets in patients with chronic renal failure. *Chem Pharm Bull* 1984; **32:** 4497–4505.
10. Abiko T, Sekino H. Synthesis of Six Common Amino Acid Sequence Fragments of Thymosins $\beta_4$, $\beta_8$ and $\beta_9$ and Determination of their Effects on the Low E-rosette Forming Cells of Lupus Nephritis Patients. *Chem Pharm Bull* 1984; **32:** 228–236.

11. Abiko T, Sekino H. Synthesis of Calf Thymosin $\beta_4$ Fragment 16-38 and its Effect on the Impaired Blastogenic Response of T-lymphocytes of a Uremic Patient with Pneumonia. *Chem Pharm Bull* 1987; **35**: 3757–3765.
12. Maeda M, Kawasaki K, Watanabe J, Kaneko H. Amino Acids and Peptides X. Leu-enkephalin analogues containing a fluorinated aromatic amino acid. *Chem Pharm Bull* 1989; **37**: 826–828.
13. Abiko T, Sekino H. Synthesis of a Thymosin $\beta_4$-like Peptide. Deacetyl-thymosin $\beta_4^{Xen}$ and its restorative effect on depressed lymphocyte blastogenic response to phytohemagglutinin (PHA) in uremic patients. *Chem Pharm Bull* 1989; **37**: 2467–2471.
14. Abiko T, Sekino H. Synthesis of a Thymosin $\beta_4$-like Peptide, Thymosin $\beta_9^{Met}$, and its Effect on Low E-rosette-forming Lymphocytes of Lupus Nephritis Patients. *Chem Pharm Bull* 1990; **38**: 3201–3204.
15. Yajima H, Kiso Y. Studies on Peptides XXX. Trichloroethyloxycarbonylhydrazine. *Chem Pharm Bull* 1971; **19**: 420–423.
16. Touranie JL, Navarro J, Corre C, Traeger J. Inhibitory Effect of Medium Sized Molecules from Patients with Renal Failure on Lymphocyte Stimulation by Phytohemagglutinin. *Biomedicine* 1975; **23**: 180–184.

**Received 10 December 1991; accepted in revised form 22 January 1992**
